# Parents’ Acceptance of Educational Technology: Lessons From Around the World

**DOI:** 10.3389/fpsyg.2021.719430

**Published:** 2021-08-30

**Authors:** Eliana Maria Osorio-Saez, Nurullah Eryilmaz, Andres Sandoval-Hernandez

**Affiliations:** Department of Education, University of Bath, Bath, United Kingdom

**Keywords:** parental engagement, use of technology, school closures, home learning environment, technology acceptance model

## Abstract

One of the long-term lessons from the school closures due to the global pandemic COVID 19, is that technology and parental engagement are the best levers to access education so as to bridge the achievement gap between socially disadvantaged children and their peers. However, using technology is not as simple as bringing equipment into the school and home and initiating its usage; these are just the first steps into a more complex and ambitious achievement of using technology as a catalyst for a shift toward new learning models in remote and hybrid settings. A theoretical framework based on the theory of acceptance and use of technology and social cognitive learning theory was used to analyse data from a survey completed by 4,600 parents from 19 countries during the national lockdowns in 2020. Regression models and thematic analysis of open-ended responses were employed to identify factors that contribute to parental acceptance and use of technology in support of their children’s learning. Our results show that parents are more engaged in children’s learning when well-structured technological tools are provided or suggested by schools, and when parents are socially influenced by the opinions of other parents, teachers, children, the general public, relatives, etc. Conversely, they are less engaged when they perceive the technological tools to be challenging and beyond their knowledge or skills. The study’s findings have practical implications for governments and school leaders, who need to be aware of the factors likely to determine the use of technology at home and take action to meet parents’ needs when using technology to support learning.

## Introduction

On average, almost half of 15-years-old across Organisation of Economic Cooperation and Development (OECD) countries are enrolled in schools where the headteacher reported that an effective online learning support platform was available (Ikeda, 2020). The picture is similar when it comes to the availability of adequate professional resources for teachers to learn how to use available digital devices, with 65% of them having access to this kind of resource across OECD countries (Ikeda, 2020). However, the support parents receive to help their children in using the same technologies to learn at home remains unexplored (Garbe et al., 2020; Müller and Goldenberg, 2021).

The UNESCO global monitoring system of school closures caused by COVID-19 showed that in April 2020, over 1.6 billion learners in 194 countries were affected. Four months later, schools remain closed in 105 countries. This means that, during this period, approximately 12 million parents around the world faced the challenge of educating their children at home. School closures have increased the existing achievement gap. Evidence from different studies around the world suggests children have made less academic progress compared with previous year groups and that there is a large attainment gap for disadvantaged students, which seems to be getting wider (Maldonado and De Witte, 2020; Domingue et al., 2021; Engzell et al., 2021; Kogan et al., 2021; Pier et al., 2021). This represents a once in a lifetime opportunity to unpack the lessons that can be learnt from the impact of this global emergency. Provision of devices and access to the Internet are key steps, but not the only ones. Working closely with parents to help them to use technology to support their children’s learning is critical as well. Combining parental engagement and use of technology is the best strategy in mitigating both the short and longer-term impacts of COVID-19, where years of progress made in education around the world are now under threat (Cruddas, 2020; Novianti and Garzia, 2020; OECD, 2020).

There is limited information on parents’ ability to enhance their skills and the factors that facilitate their engagement with children’s learning when utilising the existing online learning support platforms chosen by schools. Available data is limited and mainly describes the provision of devices, access to the internet (UNICEF, 2020) and concerns about parents’ ability to keep their children safe online (OFCOM, 2020).

This study is aimed at identifying the factors associated with fostering parental acceptance and use of technology to support their children’s learning in 19 countries. Social cognitive learning theory (SCLT) (Bandura, 1999) and the theory of acceptance and use of technology (TAMs) (Venkatesh and Davis, 2000; Venkatesh et al., 2003; Venkatesh and Bala, 2008; Abdullah and Ward, 2016) are used to explain how parents receive and use technology to support children’s learning. Under SCLT, a socially appropriate outline for explaining how parents approach technology is proposed, while TAMs explain what factors influence parental acceptance and technology use.

This paper is organised as follows: section two provides a review of the literature relating to parental engagement, home learning environment, social-cognitive learning theory, and the theory of acceptance and use of technology. Section three presents the exploration of the current international data on parental engagement and acceptance and use of technology. Section four presents the research questions that guide the present study. In Section five, the method employed for gathering the data is explained, whilst Section six presents the results derived from this study. Section seven discusses the study’s findings and the last section concludes with recommendations for policy and future research.

## Literature Review

### Parental Engagement With Children’s Learning

Evidence from research has shown parental engagement in children’s learning is critical to student success (Fan and Chen, 2001; Desforges and Abouchaar, 2003; Jeynes, 2005, 2007, 2012; O’ Brien et al., 2014; Purcell-Gates et al., 2014). O’ Brien et al. (2014) found that children whose parents participated in intervention programmes experienced substantial growth in language and literacy. These findings support the idea that parents are the best partners to close achievement gaps (Goodall, 2017). Hence, parents as equal partners, with a voice and an active presence, support learning and not only homework or the curriculum.

Yet, a consensus of what parental engagement means is still problematic as it has many definitions. According to Kim, parental engagement refers to parents’ involvement in their children’s lives in order to enhance their outcomes (2009, p. 89). As such, parental engagement is not just involvement in or support of the school, but also, helping with learning (Goodall and Montgomery, 2014). This perspective entirely changes the traditional role parents have played whereby they are part of a limited partnership that supports the schools’ goals. In the present study, Goodall and Montgomery’s (2014) continuum, which charts parental involvement to parental engagement, is used as a framework in measuring parental engagement with children’s learning.

To better understand what parental engagement is and how it is operationalised, let us start by defining parental involvement. According to Latunde (2017), there are two types: traditional and non-traditional. Traditional forms of parental involvement include helping with school homework, attending parents’ evening and social events (Goodall, 2013, 2018; Torre and Murphy, 2016; Watt, 2016) and volunteering in the classroom (Lewis et al., 2011). Under this perspective, parents are treated as peripheral to education (Pushor, 2007), which places the school in a privileged place of having expertise and power (Latunde, 2017, p. 10), and parents as having a minor impact on student educational outcomes (Jeynes, 2005).

Non-traditional definitions of parental involvement have emerged more recently and have broadened the spectrum of parental engagement. Parental engagement includes, among other activities, parents providing moral and emotional support, reading with their children, promoting and supplementing learning, following and supporting their children’s learning interests, modelling learning, modelling resilience and creating learning environments (Latunde, 2017). The premise is that learning is a broad concept, one beyond the school curriculum and not limited by the school walls, with parents playing an active role in it.

Parental engagement shares a powerful connection with children learning at home and it is a strong predictor of children’s achievement (Harris and Goodall, 2008; Harris et al., 2009). Parents not only support the school curriculum but learning in all its forms and feel empowered enough to work alongside the teaching staff, suggesting new ways to approach tasks and solving problems, as well as leading children’s learning processes.

Studies have proven that parental behaviours and attitudes toward learning impact upon children’s learning. That is, parental engagement is essential for improving educational outcomes (Melhuish et al., 2008; Jeynes, 2012, 2014a,b; Huat See and Gorard, 2015; Karemaker et al., 2017). Studies in the field have been predominantly small scale. They have used inconsistent parental engagement definitions focused on participation in school-based activities, rather than engagement with children’s learning. Consequently, the field has had to rely on a few large-scale research studies providing evidence of a relationship between specific parents’ behaviours, strategies and factors that underpin their engagement.

### Home Learning Environment

Parental engagement happens long before schooling, and it is one of the elements of the home learning environment. Homes are not only the place where parents cover basic needs, such as affection, safety and survival. They pass on knowledge and capital that children use on a daily basis to their benefit (Bjorklund et al., 2002; Bornstein, 2006). Homes become transformed into learning environments to educate young people for successful adjustment to cultural, physical, social and technological challenges.

**FIGURE 1:**
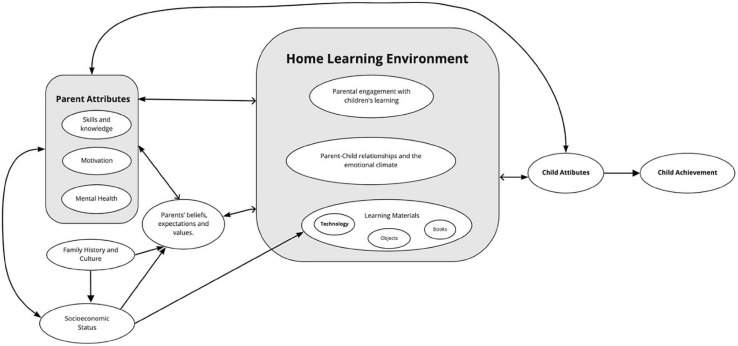
Conceptual model of home learning environment adapted from Dearing and Tang (2010, p. 131).

Scholars have presented frameworks to determine the elements and relationships in the home learning environment. For instance, Bornstein (2006) and Bradley and Corwyn (2004) have presented frameworks that have focused on cognition, language, and socio-emotional skills, whilst others have centred their attention on the effect of the home learning environment on achievement (Hess and Holloway, 1984; Brooks-Gunn and Markman, 2005; Melhuish et al., 2008). Home learning environment frameworks are diverse, but they share many common factors that contribute to children’s success. An alternative framework focusing on what happens at home and not at school has been proposed by Dearing and Tang (2010, p. 131). In the present study, we have employed and adapted three elements presented by Dearing and Tang (2010) to define home learning environments; (1) parental engagement with children’s learning, (2) the parent-child relationship and emotional climate that favours learning, and (3) learning materials, with an emphasis on technology (see Figure 1).

The first characteristic of the home learning environment is parental engagement. It was explained in the previous section as referring to activities that parents and children share that stimulate learning. It also involves the actions that parents undertake to enhance their skills to support children’s learning. The second characteristic is the parent-child relationship and emotional climate that favours learning. It refers to a positive home atmosphere that parent and child create together (Dearing and Tang, 2010). This is mediated by parenting style (Maccoby and Martin, 1983) and the store placed on education at home (Francis and Archer, 2005), which depends on parents’ beliefs, expectations, and values.

The last characteristic is learning materials. Children learn through interaction with others and objects (Piaget, 1950, 1951, 1981). Research suggests that material resources are necessary for brain development, and the absence of objects limits neural growth (Blakemore and Frith, 2005). In a study with children raised as deprived orphans, Rutter et al. (2004) found that learning objects played a crucial role in recovery from cognitive deficits. Studies have evidenced children’s access to books as a predictor of literacy achievement (Clark, 2011). To sum up, learning materials at home are effective; however, these materials have to match the developmental stage.

Technological devices and software are also critical learning materials in the home and parents play an active part in the development of a digital learning environment (Baruch and Erstad, 2018). Digital literacy concerns have been a key focus of national education policy agendas (Mossberger et al., 2007; Selwyn, 2016, 2017; Livingstone et al., 2018; Livingstone and Blum-Ross, 2019, 2020). Global initiatives from across both the private and public sectors have been implemented to provide access to up-to-date educational technology to narrow the digital divide (Bozkurt et al., 2020; Van Dijk, 2020; Pangrazio and Sefton-Green, 2021) and enable the development of the necessary skills for facing the challenges that technology brings to schools and workplaces. These initiatives have involved the provision of equipment and access to the internet. They have been reproduced in different countries with similar aims and target populations of school-age students. Countries and regions, including the United States, Australia, Panama, Uruguay, Costa Rica, Colombia, Europe, and the United Kingdom have launched a range of small- and large-scale projects providing technology in schools (Zheng et al., 2016). Their experiences vary, with there being mixed evidence on the impact of these projects due to infrastructure, investment, school leadership, teacher training and home involvement during the implementation.

1:1 Technology not only impacts on how children learn and interact in schools, but also transforms the dynamics at home and potentially determines whether learning is taking place (Lei et al., 2011). Devices in the home increase access to resources and information (Zucker and McGhee, 2005). That is having a personal device at home not only facilitates academic-related tasks, but also increases opportunities to engage with online/offline games, social media (Lei et al., 2011), family interactions and potentially develop skills in parents that enable them to better support their children. The provision of technological devices and access to the internet is the first step toward using technology to support learning at home. Before using technology as part of the learning routine at home, parental acceptance of it is crucial. Parents decide which devices to purchase, how many devices per child, how many hours to use them and the level of restrictions applied to their usage. These decisions are based on parents’ attitudes toward the use of technology, which are based on their beliefs, expectations, values, parenting style, and the social influences they are subject to Venkatesh et al. (2003).

How parents accept and use technology is explained by two intertwined theories: Social cognitive learning theory (SCLT) (Bandura, 1977, 1986, 1997, 1999) and the theory of acceptance and use of technology (Davis, 1985; Venkatesh and Davis, 2000; Venkatesh et al., 2003; Venkatesh and Bala, 2008; Abdullah and Ward, 2016). SCLT postulates a socially fitting framework for explaining how parents approach technology through observations, interactions and discussions with their children, relatives, other parents, and teachers (Livingstone and Blum-Ross, 2020). While the theory of acceptance and use of technology explains what influences parental acceptance and use.

Social cognitive learning theory suggests that human beings learn both behaviours and cognitive strategies from observing how others behave and that these assets can be acquired without being directly reinforced (Green and Piel, 2009). Observing becomes a powerful tool for learning new information and ideas that lead to the development of behaviours (Bandura, 1999) and attitudes toward the acceptance and use of technology. For instance, (a) parents observing other parents using certain applications for supporting learning at home; (b) parents following a teacher’s recommendation on a specific website that can boost students’ performance in maths or (c) parents observing their children troubleshooting a device at home. A positive outcome in this observation process might lead to a change of behaviour in the parents and how they relate to technology.

The self, environment and behaviour are the domains of SCLT. These are represented in external and internal social reinforcement, social influence (Venkatesh et al., 2003), past experiences and self-efficacy (Bandura, 1978, 1997), all of them playing a vital role in a reciprocal interaction. The first element, external and internal social reinforcement, influences the way parents acquire and maintain behaviour. For instance, parents use emails as the primary way to communicate with teachers because the school has suggested it, whilst also receiving information from other parents on alternative forms of approaching teaching staff, such as phone calls, text, and WhatsApp messages.

The second element, social influence (Venkatesh et al., 2003), pertains to the degree to which a subject perceives it essential to others that they perform an action or undertake a change in behaviour. It refers to what is accepted as the group norm or group “subjective culture that the individual has made with others, in specific social situations” (Triandis, 1979, p. 210). In this regard, accessing different perceptions and opinions might be a powerful source of inspiration for how parents welcome and use technology at home to support children’s learning. The third element is the parent’s past experiences. That is, parents’ past experiences influence whether the action will occur or not; they shape whether a parent will join in specific behaviour as well as explaining the reasons and expectations that reinforced that decision. These will be heavily influenced by their own experience in schooling, previous experiences dealing with technology at the workplace and/or daily life.

The last element is self-efficacy (Bandura, 1977, 1978), which refers to a parent’s beliefs in her/his ability to influence her/his child and the environment in ways that will foster the child’s development and success (Ardelt and Eccles, 2001). Parental self-efficacy is influenced by parents’ specific capabilities, confidence as well as other individual factors and environmental factors that may act as barriers or facilitators. Self-efficacy involves parents’ conviction that technology can be used as a tool to enhance learning; however, to reach that conviction, they need to welcome or accept technology as that powerful tool.

The process of technology acceptance has been explained by several theories: diffusion and resource dependence theory (Pfeffer, 1982), innovation adoption theory (Rogers, 1983, 2003); technology and social inclusion (Warschauer, 2004; Alonso Cano et al., 2010; Ceretta and Canzani, 2016; Cobo and Rivera-Vargas, 2018); and the technology acceptance model (TAM) (Davis, 1985; Venkatesh and Davis, 2000; Venkatesh et al., 2003; Venkatesh and Bala, 2008; Abdullah and Ward, 2016). In this paper, we use the TAM to measure parental acceptance and technology use. A key selection criterion was how TAM provides insight on what factors influence parental acceptance and technology use.

**FIGURE 2:**
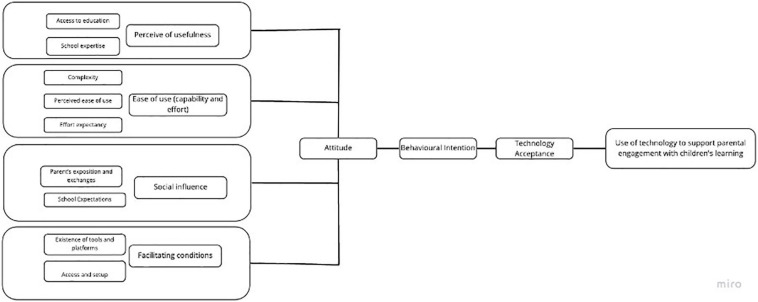
Hypothesised model for the acceptance and use of technology to support parental engagement with children’s learning.

Technology acceptance models models have been widely used in previous studies looking at how technology is accepted and used by students, educators, and employees. Previous studies, such as those of King and He (2006); Šumak et al. (2011), and Abdullah and Ward (2016), have shown that TAM is the most commonly applied and robust theory in existing research for understanding users’ acceptance of technology in a variety of contexts. Since its appearance in 1985, the original TAM has been adapted and complemented with different factors or variables. In Davis’s (1985) original model, three factors were introduced, all of them in order to reflect the context of the application. Four main factors determine an individual’s acceptance and use of technology: perceived usefulness, ease of use (capability and effort), social influence (Venkatesh et al., 2003), and facilitating conditions (Triandis, 1977). We investigated the last three factors to explore parental acceptance and use of technology as a preliminary step to enhance their skills, build competencies and facilitate their children’s learning (see Figure 2).

Perception of usefulness (Venkatesh and Davis, 2000) is an essential factor, for it determines whether the technology is useful for supporting learning. During the national lockdowns, the only way to access education was via different forms of technology: online platforms, apps, informative emails, WhatsApp messages and calls, phone calls, videoconferencing, printed materials, etc. So, technology was already perceived as useful and essential for accessing education. Additionally, the selection of learning management systems lies with the schools, governments, or local education authorities. That is, these organisations are those who decide which educational technology should be acquired, purchased and given to families. There are even some cases where schools not only select the educational technology to use in terms of the software, but also which device is allocated to students. For instance, in the Learning Foundation 1:1 programme in the United Kingdom, schools are tasked with choosing which technological devices parents should purchase (Learning Foundation, 2021). Consequently, the perception of usefulness is outside of parents’ control in the decision-making process, and for that reason was not explored in this study.

Ease of use (capability and effort) is explained as parents’ perception of how easy or difficult it is to use educational technology given their abilities (Davis, 1989). It includes positive and negative factors, such as complexity [negative] (Rogers, 1983, 2003; Prensky, 2005; Goodyear and Carvalho, 2019; Haaranen et al., 2020), perceived ease of use, effort expectancy, and past experiences and self-efficacy (Bandura, 1978, 1997). Social influence includes parent’s exposure to, exchanges with, and access to the perceptions of others (parents, teachers, children, the general public, and relatives, etc.) in the use of the educational technology, including how one is perceived by others (Venkatesh et al., 2003). Social influence also refers to the use of educational technology, regarding whether it is indispensable for the completion of tasks via online platforms, apps, and networks, etc. Facilitating conditions pertain to the systemic or situational factors that affect the access and ability to use the educational technology (Triandis, 1977; Venkatesh and Bala, 2008). In the present study, facilitating conditions specifically refer to the acquisition and access granted to children and parents to well-structured learning management systems or apps selected by the school. Investigating the three above factors can provide understanding of parents’ attitudes toward the acceptance and use of technology to support their children’s learning.

## International Data on Parental Engagement and Acceptance/Use of Technology

### International Large-Scale Assessments

Internationally comparable data on parental engagement and parental acceptance and use of technology in education is extremely limited. Data from the OECD and the United Nations Children’s Emergency Fund – UNICEF data present a limited approach to parents’ participation in their children’s education. The Programme for International Student Assessment PISA, for example, only presented findings from their parental involvement questionnaire distributed in 15 countries during 2009, 2012, 2015, and 2018. Moreover, in the PISA questionnaire parental engagement was relegated to some retrospective questions, and only captured attitudes toward reading, self-reading time, parents’ texts preferences and, in 2018 only, parents’ involvement in online extended reading activities.

Furthermore, when exploring parents’ attitudes toward using technology to support children’s learning, international studies like PISA and the Trends in International Mathematics and Science Study – TIMSS did not establish a clear theoretical background. Whilst a review of the PISA Assessment Framework (OECD, 2019) shows that some references were made to theories developed by authors, such as Bryk (2010); Chapman et al. (2012), and Klauda (2009), no explicit reference was made to indicate what concepts from which theories were underlying the items used to create the corresponding items and scales.

A similar approach is taken in the design and analysis of the Multiple Indicator Cluster Survey (MICS) carried out by UNICEF (UNICEF, 2020). It is designed on the basis of the identified needs for the national and subnational monitoring priorities. Currently, the sixth and largest round of surveys (MICS6) is being undertaken, with the largest numbers being in Europe and Central Asia (17 surveys in total) and West and Central Africa (12 surveys). More details are provided in Table 1.

**TABLE 1:** MICS surveys by phase and territory.

Phase	South Asia	East Asia/the Pacific	Europe/Central Asia	Eastern/Southern Africa	Middle East/North Africa	West/Central Africa	Latin America/the Caribbean	Total per region
1	7	8	5	13	10	18	2	63
2	4	7	10	11	13	14	7	66
3	1	6	13	6	8	13	6	53
4	5	9	10	7	7	12	10	60
5	6	7	9	6	4	11	9	52
6	7	10	17	5	6	12	10	67
Sum total	30	47	64	48	48	80	44	**361**

This extensive survey has involved seven progressively updated versions or rounds. MICS collects data to identify key indicators used to assess children and women’s situations across the world. It presents disparities in the home learning environment across and within countries. The inequalities are represented in terms of access to learning materials, such as books and technological devices. The findings of MICS6 provide only a limited measurement of parental engagement with children’s learning, considering only two factors: parents supporting homework and the number of reading books at home. The surveys have also explored parents’ IT skills outside of the educational context (UNICEF, 2020); however, this information is not linked to children’s learning.

### Recent Empirical International Studies

Between 2018 and 2019 a series of reports were published as part of the UNESCO-Fazheng project. The reports described fourteen case studies from twelve countries. These case studies were governments, ministries of education and school-led initiatives on best practices in mobile learning. The findings from the initiatives led by governments and ministries of education in Uruguay (Cobo and Rivera-Vargas, 2018); Croatia (Smoljo and Korda, 2019); the republic of Korea (Lim and Kye, 2019) and Rwanda (Wallet and Kimenyi, 2019) presented few or no impactful activities regarding parental engagement with children’s learning. In these initiatives, parents seemed to perform a secondary role in the implementation of mobile learning. On the other hand, initiatives led by individual schools or groups of schools in China, Brazil, the United Kingdom, Russia, and Portugal showed that parents played an active role while dealing with technology to support learning. These countries presented evidence of the impact of shared visions, partnerships between home and school, and having a training programme to make parents part of the initiative.

In the same studies, China reported findings where a school assessed parents’ digital skills according to their job or occupation and presented opportunities within the school for parents to develop digital skills and create online resources for school platforms (Su and Li, 2019). Parents were also frequently invited to participate in events, meetings, and activities with their children (Yu et al., 2019). Related results were found in Portuguese and Spanish schools, where researchers observed increasing interest amongst parents in the school’s activities and pupils’ motivation to attend both extracurricular activities and regular lessons as well as a lowering dropout rate (Hinostroza et al., 2019; Lima and Tulivuori, 2019). According to these reports, school-led initiatives seem to be more successful in engaging parents in the use of technology to support learning. Some studies imply this is due to a shared vision regarding the use of technology (Uvarov et al., 2019; Yu et al., 2019) and constant school-home communications (Barbosa et al., 2019). No schools in any of the two models of implementation reported factors associated with how parents accept or use technology to support children’s learning.

## Research Questions

Many studies have explored the impact of COVID-19 on employment, management of the pandemic, the economy, mental health and student achievement and teaching. However, very few have focused on how parents are coping with home-schooling; the strategies they are using, the synergies they are developing, the partnerships they are establishing and the challenges and opportunities educational technology has opened to them under the current circumstances. Studies focusing on parents’ perspectives possess a narrow theoretical basis and their scope is limited to how parents are supporting the school agenda. This empirical study was designed to extend the existing body of knowledge to explore the factors likely to shape parents’ acceptance and use of technology to support their engagement with their children’s learning.

The following questions were proposed to address the above-identified research gaps.

• To what extent does parents’ acceptance/use of technology influence their engagement with children’s learning?

Three specific sub-questions guide this study:

• To what extent do others’ opinions influence the use of technology in supporting parental engagement with children’s learning?
• To what extent does the use of existing school technology impact upon parental engagement with children’s learning?
• To what extent does the effort needed to use online tools impact upon parental engagement with children’s learning?

## Materials and Methods

### Data

The data from this study stems from the International COVID-19 Impact on Parental Engagement Study (ICIPES) (Osorio-Saez et al., 2020). ICIPES was a joint effort in 23 countries to investigate the ways in which parents and caregivers engaged with their children’s learning during the period of social distancing arising from the global COVID-19 pandemic.

Data was collected using an online survey with a total sample of 4,658 parents/caregivers of children between 6 and 16 years old, living with their child. Children were between grade 1 and 13, which represents between 1 and 13 years of schooling, counting from the beginning of Level 1 of the International Standard Classification of Education-ISCED (UNESCO, 2011). The survey was administered by the University of Bath team using the JISC Online Survey Tool (Osorio-Saez et al., 2020). All respondents gave their informed consent and the research collaborators only had access to the data after it had been fully anonymised.

The four main domains explored in the questionnaire were: Parental engagement with children’s learning, School support for parents and children, Home-schooling and family life balance and Parental acceptance in the use of technology. The full version of the Osorio-Saez et al.’s (2020, 2021) background questionnaire can be found in the ICIPES User Guide. In this paper, data from two domains were used: Parental engagement and Parental acceptance in the use of technology, as shown in Table 2. In addition to the data collected from the Likert scale responses, the research design also included the following six open-ended questions:

**TABLE 2:** Themes included in the questionnaire.

Domains in ICIPES, 2020	Subdomains	Items	
		Likert scale questions	Open-ended questions
Parental engagement with children’s learning (Kim, 2009; Goodall and Montgomery, 2014)	Parental engagement	5	6
Parental acceptance in the use of technology	Facilitating conditions (Triandis, 1977)	3	
	Social influence (Venkatesh et al., 2003, p. 451)	4	
	Ease of use (Capability and effort); Complexity (Rogers, 1983, 2003; Prensky, 2005; Goodyear and Carvalho, 2019; Haaranen et al., 2020)	10

(1) Tell us more about the school’s support during home-schooling throughout the COVID-19 lockdown period.
(2) Are you teaching your child at home? (Taking the time for sitting and explaining the topics and activities to them) Why not?
(3) Tell us more about how you get prepared yourself to support your children’s learning.
(4) Tell us more about how you teach your children at home.
(5) Tell us more about the activities you and your children do together during the lockdown period.
(6) Tell us more about how confident you feel dealing with technology to support your children’s learning.

The descriptive and augmented texts from the answers to the above questions were used to explain the quantitative findings in this study.

Even though we received responses from 23 countries, the information of four was omitted due to a low response rate. So, we used a final sample of 4,600 parents residing in 19 countries. More detailed information about each country’s respondents can be seen in Table 3.

**TABLE 3:** Participant demographics.

Countries	Number of Participants
Ethiopia	171
Ghana	142
Tanzania	58
China	217
Japan	159
Italy	517
Turkey	78
United Kingdom	191
India	54
Pakistan	45
Sri Lanka	199
Chile	1,597
Colombia	94
Costa Rica	155
El Salvador	83
Honduras	246
Mexico	244
Uruguay	61
United States	289
*N* =	4,600
**Area**	
Urban	3,725
Rural	747
Other	128
**Family composition**	
Living with the father/mother of the child	3,626
Living with a partner who is not the father/mother of the child	275
Raising a child without a partner	591
Other	108
**Parent age**	
Under 18 years old	32
18–24	47
25–34	740
35–44	2,232
45–54	1,329
55–64	188
65–74	30
75 or older	2
**Gender**	
Female	3,529
Male	1,071

### Variables

The main dependent variable was parental engagement with children’s learning (ENG_Scale). This scale was constructed by the Osorio-Saez et al.’s (2020, 2021) research team using five items and is included in the international dataset. Parents were asked to what extent they agreed with the following statements: Q21_2 *I follow my ideas about what my children need to learn*, Q21_3 *I mix my own ideas with the school’s plan on what my children need to learn*, Q22_2 *I list and prepare the activities myself before developing them with my child(ren)*, Q22_3 *My children and I have a set home-schooling timetable*, Q22_6 *I develop with my children spontaneous learning activities not necessarily school-related such as cooking, woodwork, online games, physical activities, etc*. The response options were organised on a five-point Likert scale, with the categories “Always,” “Often,” “Occasionally,” “Rarely,” and “Never.”

The leading independent variables were social influence (four items), facilitating conditions (three items) and ease of use (capability and effort/complexity) (10 items). Parents were asked about the frequency with which they carried out different activities using technology (response options: Always, Often, Occasionally, Rarely, and Never), and how confident they felt doing so (response options: not at all confident, slightly confident, moderately confident, quite confident, and extremely confident). More information about the variables can be found in the ICIPES Technical documentation (Osorio-Saez et al., 2021).

The other independent variables included in the analysis can be organised into two groups, namely characteristics of the family and characteristics of the students. The following variables are part of the first group: location (urban/rural), parental gender (male/female), parent years of schooling, parent age (in years), the number of children in the household and family socioeconomic status. In the second group, we have the following variables: child’s gender (male/female), and child’s years of schooling. The purpose of including these variables is that they are theoretically associated with the outcome of interest (parental engagement), so we use them here as control variables. Table 4 provides detailed descriptive statistics for the variables used in this study.

**TABLE 4:** Descriptive statistics for the variables used in this study for all countries.

	Minimum (min)	Maximum (max)	Mean	Standard deviation (SD)
Dependent variable				
Parental engagement	−1.958	2.729	0	1
Independent variables				
Facilitating conditions	−2.778	1.757	0	1
Social influence	−2.936	1.408	0	1
Effort/complexity	−2.751	1.803	0	1
Socioeconomic status	−2.356	4.003	0	1
Location (0 = Urban, 1 = Rural)*	0	1	0.186	0.392
Parent gender (0 = Female, 1 = Male)*	0	1	0.232	0.422
Parent schooling	0	25	15.43	3.754
Parent age	0	7	3.19	0.864
Child’s gender (0 = Female, 1 = Male)*	0	1	0.504	0.500
Child’s years of schooling	0	14	5.043	3.221
Children in the household	0	10	1.309	1.449

*The column titled Mean represents the proportion of the cases in the category 1.

Socioeconomic status (SES) was constructed using the following questions. Q5: What do you do in your main job? (e.g., teach high school students, help the cook prepare meals in a restaurant, manage a sales team). This was an open question that was recoded into an ordinal variable following the list of occupations described in the one-digit International Standard Classification of Occupations (ISCO). Q7: In a normal month, what is your total household income? This variable was recorded by grouping the income level reported in deciles of income within each country. Q13N asked: How many usable devices are there in the house? (Smartphones, tablets or iPads, laptops, and desktops). Q14: How many computers per child have you got at home?

### Analytical Strategy

The main data analysis method used was Ordinary Least Square (OLS) regression – a technique used to define the line of best fit for a set of data – with country fixed effects (for the 19 countries). We fitted three regression models to examine whether and to what extent the three factors included in the TAM [facilitating conditions, social influence, and ease of use (capability and effort/complexity)] predict parental engagement with children’s learning. As aforementioned, the dataset used in our analyses included data from 19 different countries. To account for the cluster (country) dependency, and following previous international comparative studies in educational research, a dummy variable was included for each country (Chudgar et al., 2013; Zhou, 2014; Gumus and Bellibas, 2016). The goal of using a country dummy variable was to control for variations in parental engagement that took place due to the differences among countries that are not included in our set of independent variables. In other words, the use of a country dummy variable can account for variations in parental engagement due to the factors specific to each country. Each regression model was fitted with this country effect, except for the first one, which was the base model.

The analysis began by fitting a model that included only the country variables for a country. This model was used to estimate the percentage of the total variation in parental engagement that was accounted for just by country effect. The following is the equation for Model 1.

(1) $$\mathrm{Parental}{\mathrm{Engagement}}_{ij}=\beta_{ij}+e_{ij}$$

The second model (2) investigated the relationship between the parental technology acceptance variables [facilitating conditions, social influence, and ease of use (capability and effort/complexity)], with the country effect controlled for. When compared with the first model, this second model provided us with an estimation of how much variation was accounted for by our variables of interest [facilitating conditions, social influence, ease of use (capability and effort/complexity)] beyond the country effect.

Lastly, in addition to our interest variables, the third model (3) examined this relationship controlling for family and children’s characteristics, as well as for the country effect. When this model was compared with the second one (2), it enabled the estimation of how much variation was accounted for by the control variables beyond our variables of interest and the country effect. The following are the equations for Models 2 and 3, respectively:

(2) $$\mathrm{Parental}{\mathrm{Engagement}}_{ij}=\beta_{1j}+\beta_{1}\mathrm{Facilitating}{\mathrm{conditions}}_{ij}+\beta_{2}\mathrm{Social}{\mathrm{influence}}_{ij}+\beta_{3}{\text{Effort}}_{ij}+\beta_{4}{\text{SES}}_{ij}+C_{j}+e_{ij}$$

(3) $$\mathrm{Parental}{\mathrm{Engagement}}_{ij}=\beta_{1j}+\beta_{1}\mathrm{Facilitating}{\mathrm{conditions}}_{ij}+\beta_{2}\mathrm{Social}{\mathrm{influence}}_{ij}+\beta_{3}{\text{Effort}}_{ij}+\beta_{4}{\text{SES}}_{ij}+\gamma {\left(\text{Parental}\right)}_{ij}+C_{j}+e_{ij}$$

For the quantitative and qualitative analysis, the lead researcher created an analysis codebook, which was informed by the study’s conceptual framework (Plano Clark, 2010; Stentz et al., 2012). Four categories were used to classify and summarise the qualitative data reflecting the original three inquiry topics [facilitating conditions, social influence, and ease of use (capability and effort/complexity)] and any new themes arising after reading the survey responses. Data were extracted manually from survey responses and summarised into four charts, three named after the variables of interest in this study and the fourth one named “other findings.” Using this data reduction and display strategy, the researchers examined the accounts of all respondents within the common thematic framework (Braun and Clarke, 2012).

Then, the results of the quantitative and qualitative analyses were compared, with the emergent themes being matched with the regression models’ results and named after the dependent and independent variables. This data added depth to the analyses, and it was used to suggest possible underlying mechanisms to explain the quantitative patterns.

The use of research collaborators during the qualitative analysis led to confirmability and consolidation of the resulting themes. Any discrepancies were discussed until an agreement was reached.

## Results

In this section, the inferential results from the regression models are discussed in tandem with the qualitative data to provide a fuller understanding. Three multiple regression models with country fixed effects were employed to examine to what extent parents’ acceptance and use of technology impact parental engagement with children’s learning. The results of each model are detailed below.

In the first instance, an unconditional model with the country fixed effect being controlled for was estimated. The results indicate that the differences among countries only accounted for about 8% of the total variation in parental engagement (see Table 5). In addition to the country fixed effects, the second model was established to investigate to what extent parents’ acceptance and use of technology and SES can predict parental engagement with children’s learning. The results show that the facilitating conditions, social influence, and ease of use (capability and effort/complexity) are all significant predictors of parental engagement with children’s learning (see Table 5). This model accounts for an additional 18% of the total variation in parental engagement. The third model investigated the extent to which the parents’ acceptance and use of technology, predicts parental engagement with children’s learning, whilst controlling for several family and children characteristics as well as the country fixed effect. In other words, it allowed us to estimate the net relationship between our variables of interest and parental engagement. This model explains just an additional 2% of the variation in parental engagement (see Table 5). The results for each of the variables of interest are provided in the next section.

**TABLE 5:** Parents’ acceptance and use of technology predicting parental engagement with children’s learning.

	Model 1	Model 2	Model 3
	**Parental engagement (With dummy)**	**Parental engagement (With dummy)**	**Parental engagement (With dummy)**

Facilitating conditions		0.135*** (6.569)	0.121*** (5.513)
Social influence		0.465*** (29.516)	0.452*** (26.936)
Effort		−0.311*** (−16.026)	−0.300*** (−14.380)
Socioeconomic status			0.010 (0.559)
Location			−0.028 (−0.842)
Parent gender			0.159*** (4.497)
Parent schooling			−0.001 (−0.346)
Parent age			0.060** (3.210)
Childs’ gender			−0.003 (−0.136)
Child’s years of schooling			0.040*** (8.512)
Children in the household			−0.006 (−0.635)
Intercept	0.054 (0.838)	−0.045 (−0.802)	−0.431*** (−3.884)
R-square	0.075	0.250	0.273
*N*	4599	4555	3931

**p < 0.01; ***p < 0.001. Notes: Sample weight is SENWT, *t* statistics in parentheses.

### The Association Between Parental Engagement With Children’s Learning and Parental Acceptance of the Use of Technology: Facilitating Conditions

A positive and statistically significant relationship between parental engagement and the facilitating conditions was revealed from the application of the regression model. Hence, the latter is a significant predictor of the former (β = 0.121, *p* < 0.001). This means that where more participating parents have been granted access to educational technology, such as Learning Management Systems, e.g., school platforms and apps, the average parents reported being more likely to engage with their children’s learning.

Parents’ strengthened desire to contribute to their children’s learning involved not only using technological devices but also the educational technology provide by the school, governments, or other non-profit organisations, as evident from parents’ responses:

*“Having an online platform helps me to organise our routine; everything is on one site, I know where the activities are, and I just need to follow the sequence. I also can check how my children are progressing on the curriculum.”*

*Parent from the United States*

*“Having an online platform is an advantage. Parents can download plenty of learning materials from there. There is also a Facebook group where teachers share videos and lessons. Each homeroom teacher has a WhatsApp group where relevant information is shared and also, she follows some important actions from parents.”*

*Parent from Colombia*

*“Thanks to the online platform and video lessons, I am on top of my children’s learning.”*

*Parent from Italy*

*“I was not familiar with the online platforms, except for Google. I, too, had to learn the programs, set up parent access, manage all of her sign-ins and passwords. I experienced glitches and issues and had to figure it out. I had never done Zoom or Google meet and had to learn them both personally. We had to learn a lot about technology to support my children quickly and the best part of it all is that there are so many great things that we can all continue to use whether we are face to face, hybrid, or remote.”*

*Parent from Chile*

*“My daughter’s school suggested using the platform and other apps and websites I find quite useful. I often visit the recommended websites.”*

*Parent from the United Kingdom*

In some contexts, facilitating conditions are absent due to factors such as inadequate equipment or infrastructure, high Internet costs, or the absence of an online platform for the school.

These contexts still require schools to provide education. School materials are sent home using low-tech and non-tech methods, such as photos sent through WhatsApp parent groups or printed materials collected from school.

This study asked some parents to describe their experiences with low tech or non-tech solutions:

*“My children’s school does not use an online platform; parents collect some printed materials and children complete schoolwork on paper or via WhatsApp. When completed, schoolwork is sent to the school; no specific feedback is provided after that.”*

*Parent from Ghana*

*“My child’s teacher sends a weekly message via WhatsApp; there are so many messages on that group that I get lost. Sometimes I miss important information; then I realise my child is behind on schoolwork, because I receive a phone call from his teacher.”*

*Parent from Colombia*

*“In Ethiopia, the Internet is expensive, and schools don’t have a website or platforms. Schools and we, parents, do what we can with the resources we have.”*

*Parent from Ethiopia*

*“Technology is not used much in our society, because of the lack of knowledge and infrastructure. The problem is resources. Schools know most households only have a phone and poor network, so they avoid sending online work.”*

*Parent from Tanzania*

### The Association Between Parental Engagement With Their Children’s Learning and Parental Acceptance of the Use of Technology: Social Influence

According to the results, social influence is a significant predictor of parental engagement (β = 0.452, *p* < 0.001), i.e., the direction of the relationship is positive. In other words, the more participating parents adopt and take part in at least one social network, the more the average parents reported being engaged with children’s learning.

Parents are influenced by the degree to which an individual perceives what others (parents, teachers, and the general public) believe they should use in terms of technologies to support children’s learning. The more parents are influenced in this way, the more they are engaged with their children’s learning.

Parents’ comments helped us to understand the relationship between these two analysed variables:

*“I make sense of homework after reading other parents and teachers’ comments on the Facebook group.”*

*Parent from Honduras*

*“I’ve taken inspiration from friends on social media, and I’ve used and followed YouTube videos too.”*

*Parent from Spain*

*“I ask friends and relatives for advice via social media. I sign up to Facebook groups, where I find great advice about schoolwork and fun activities with the children.”*

*Parent from Uruguay*

*“I follow the school daily plan, but children finish these activities in two hours. Hence, I must look for fun activities on Facebook. Family Lockdown is the best for finding inspiration of we what to do with school-age children.”*

*Parent in the United Kingdom*

*“Read before to ensure I can help/explain. Message teacher or friends if I need help!.”*

*Parent in Mexico*

*“I often check parents’ comments on social media.”*

*Parent in China*

*“I have started a WhatsApp group to talk to relatives in other countries to share the experience.”*

*Parent in Sri Lanka*

### The Association Between Parental Engagement With Children’s Learning and Parental Acceptance of the Use of Technology: Ease of Use (Capability and Effort/Complexity)

The results, in this case, indicate that when school technology is perceived as being complex to use, parents are less likely to engage with their children’s learning (β = −0.300, *p* < 0.001). In other words, the more parents have to make an effort to understand how to work with a particular piece of technology, the less they are engaged with their children’s learning.

The difficulties when using educational technology include complexity, perceived ease of use, effort expectancy, and self-efficacy, all of which prevent parents from engaging with children’s learning. Additionally, parents expressed concerns regarding their role, due to the lack of direction or guidelines in relation to what was expected from them, when their children were working with the school’s educational technology.

Parents’ concerns about how difficult it is to use certain pieces of educational technology are also evident from respondents’ comments:

*“I think the school platform is not that user friendly. When we try to circle or drag and drop, it’s really quite difficult to be precise. When completing cloze questions, answers are always wrong after typing all the possible answers.”*

*Parent from the United Kingdom*

*“They use Moodle in my [children’s] school, where there are some activities to complete online and others to print or watch videos. I get lost some time, for example, with the videos, after watching, I not sure what we should do: discuss about them? Write a summary?”*

*Parent in Mexico*

*“I struggle using the school platform. It is not user friendly. I don’t understand how it works.”*

*Parent in El Salvador*

*“I still don’t understand how to upload homework. We send them attached in emails to the teachers or school.”*

*Parent in Colombia*

*“I feel exhausted dealing with too many platforms. Zoom is the easiest one to use, but the one used for science and math classes, we don’t know how to use it.”*

*Parent in the United Kingdom*

*“We often send wrong answers in the maths homework, which is because we don’t know how to add some signs to the equations. It would be better if we could just work on a piece of paper and send a picture.”*

*Parent in Italy*

*“The app that we are using with the school is unfriendly and difficult to use. The school says we can print; but it not easy printing worksheets from the app.”*

*Parent from India*

### The Association Between Parental Engagement With Their Children’s Learning and the Family and Children’s Characteristics

Regarding the other control variables included in the model: parent gender, parent age and child’s years of schooling were significant in predicting parental engagement. On the other hand, SES, location, parent schooling, child’s gender, and the number of children in the household were not significant. These are interesting results, but space constraints prevent us from discussing them in detail.

Whilst SES is not a significant variable for predicting parental engagement (see Table 5), qualitative data reveals that parents in some countries expressed their concerns about not having resources or the most suitable devices for children to access education:

*“My children (4 in total) use one computer or my smartphone, taking turns This slows their learning at home and for me it is impossible to help them to complete the activities in one day.”*

*Parent in Tanzania*

*“In Ghana, Internet is expensive, so we cannot top-up the phone to share the internet every day. Therefore, no homework can be finished.”*

*Parent in Ghana*

*“Ideally, he should be working in a tablet or iPad, but we don’t have the money now. The mouse is too big for his little hands.”*

*Parent in the United Kingdom*

*“She works on an old computer that was borrowed from the school, not the best or fastest, but the only way to access school lessons.”*

*Parent in the United States*

Regarding parent gender (β = 0.159, *p* < 0.001), the results suggest that male parents are more likely to engage than female ones. Fathers commented on their engagement with learning:

*“Balancing home and work commitments has been tough… I have become my daughter’s maths teacher.”*

*Father from Chile*

*“I particularly enjoy when teachers call to find out how they are doing. Speak to the children, visited them at home and also send them work to do. I have become a fully involved father.”*

*Father from Ghana*

Parents’ age showed a weak but statistically significant association with engagement (β = 0.060, *p* < 0.01), That is, the older the parents reported as being, the more likely they were to engage with children’s learning. Whilst open-ended responses from parents describe some concerns about the relationship of their age and IT skills to support learning effectively, they also commented on what action they take to become informed:

*“I watch videos on YouTube to understand before sitting with my children to do the activities. When I was at school there were no computers, so there is a lot I need to learn.”*

*Parent from Costa Rica*

*“I call my youngest sister to troubleshoot the computer.”*

*Parent from Mexico*

*“At my age, technology is challenging, that’s why I try hard to keep up.”*

*Parent from Pakistan*

Children’s years of schooling are also positively associated with engagement (β = 0.040, *p* < 0.001). In other words, the more the years of formal schooling of the children, the more their parents tend to be engaged with their learning. Whilst the vast majority of parents, indeed, want to take part in their children learning activities, the way in which those with secondary-school aged children engage varies. In general, the qualitative data suggests that for this group of children, the engagement becomes more supportive than guiding or teaching:

*“They are old enough to tackle homework and complete their activities, but we always keep an eye on ESafety.”*

*Parent from the United States*

*“My son is 17 years old. He does not come often to me to discuss homework, but he comes to me when he wants to be sure the information online is accurate and not fake.”*

*Parent from Colombia*

## Discussion

This study was aimed at providing empirical evidence for the factors that influence parents in accepting and using technology to support their engagement. For the research, data from a survey of 4,600 parents from 19 countries collected in 2020 during the national lockdowns due to the global pandemic of Covid-19 were analysed. Three regression models were employed to identify factors that contribute to parents’ acceptance and use of technology to support their engagement with children’s learning.

Concerning parental acceptance and use of technology, our findings indicate that social influence (Venkatesh et al., 2003), facilitating conditions (Triandis, 1977), and ease of use (capability and effort) (Davis, 1985; Venkatesh and Davis, 2000) are significant determinants of such engagement. Parents perceive that the school has facilitated access to educational technology, such as learning management systems and apps; however, ease of use (capability and effort) often prevents them from engaging with technology to support learning. They report how some of the school educational technology is complex (Rogers, 1983, 2003; Prensky, 2005; Goodyear and Carvalho, 2019; Haaranen et al., 2020). This idea of complexity is explained by lack of experience in dealing with technology, the intricate look and feel of the platforms and apps as well as bugs and errors in some low-tech educational tools.

### Complexity as a Barrier

This idea of complexity as a barrier is in line with the findings of previous studies (Rogers, 1983, 2003; Prensky, 2005; Goodyear and Carvalho, 2019; Haaranen et al., 2020) that have provided evidence of a strong link between acceptance/use of technology and parental engagement. The fundamental factor in making educational technology-EdTech amiable is the design of the interface. Hence, an educational platform app and/or school website should be well structured, user-friendly, and easy to navigate.

The selection of EdTech should follow six basic steps; Review of scientific research into how people learn and the best ways to integrate technology with singular learning approaches (Bower, 2019). Assessment of the external (previous and current) users of the system (Connaway et al., 2011). Assessment of the potential institutional users, which might offer the most suitable starting point and possibilities for training and support, which is applicable to the learner, the teacher and the parent that support learning (Hinostroza et al., 2019; Lim and Kye, 2019). Pre-tests or pilots of the new system (Bossavit and Parsons, 2018; Tsivitanidou and Ioannou, 2021) to test functionalities and ensuring absence of error, crashes and reliance on other devices/elements. Revision of the quality in the embedded content. Finally, assessment of the educational compatibility (Rogers, 1983, 2003; Chen, 2011; Kemp et al., 2019) of the technology applied at all levels to achieve the expected learning outcomes.

### Social and Family Influence

From the results, it also emerged that social influence, some in the form of virtual communities (Rivera-Vargas et al., 2017) plays a role in helping parents to engage with the use of technology to support children’s learning. Social Learning Theory supports this finding. Parents value having access to other’s perceptions and opinions in scheduled and spontaneous exchanges with other parents, teachers, children, the general public, and relatives. These exchanges allow for them to self-assess their performance and role in home-schooling, voice their struggles, and help them to find answers as well as alternative ways to deal with the challenges that home-schooling imposes. In this regard, “others” support parents navigating not only in the challenges that educational technology presents, but also, as a networking mechanism so to be up to date in traditional parental involvement activities with schooling and parental engagement activities that allow their children to reach their potential. Similar findings have emerged from a recent empirical study in Australia (Ewing and Vu, 2021) and Livingstone and Blum-Ross (2020) who found that as part of their digital engagement, parents valued collaborative learning.

Other difficulties reported when dealing with technology at home are associated with the number and type of activities that are sent on a daily basis, as well as parents’ perception that some activities will work better on paper than on a screen, such as writing and spelling. Many also mentioned the lack of resources to do some of the homework such as not having a printer, a digital pen, trackpad, and an ergonomic mouse, etc.

Some clarity on parents’ role in educational technology is also imperative, moving away from the expected role of policing screen time (Livingstone and Blum-Ross, 2020) and homework. Many expressed how they make sure activities are undertaken and completed; however, when there are online resources that contextualise or extended learning, they struggle to find out what is expected from them. A framework or checklist to be distributed among schools, where they can set and self-assess their institutional strategies to help parents in dealing with technology, would be of value. This material should include the channels for advice on regulations (security and safety), channels for training (workshops, video tutorials, spaces for parental discussions, and guidelines for promoting child/parent conversations) and clear definition of the parents’ role in supporting learning. This framework might respond to the need to make educational technology more user friendly to parents, as well as facilitate open spaces for partnerships and discussions with families in relation to the selection of the most suitable educational technology according to collective experience.

### Other Findings

Other findings from this study have shown that male parents are more likely to engage with their children’s learning than female ones. This finding is consistent with previous research suggesting that even when fathers have had limited schooling, their involvement in their children’s schools and school life is a powerful factor underpinning their academic achievement (Grolnick and Slowiaczek, 1994; Nord, 1997; Gadsden and Ray, 2003; McBride et al., 2005). However, more research needs to be carried out to investigate fathers’ and mothers’ roles in dealing with educational technology. It was also found that older parents are more engaged with children’s learning. Research on how parents get engaged according to their age regarding the acceptance and use of educational technology is required.

One last finding emerged from the present research. The greater the number of years of formal schooling of the children, the more their parents tend to be engaged with their learning. Previous studies, however, have highlighted that some forms of parental involvement can be beneficial in the early years of schooling but less so in later years (Jeynes, 2007; McNeal, 2012; Patall et al., 2008). One possible explanation for this finding could lie in the parents’ concerns about screen time, online safety, and the evolution of the parents’ role in secondary school.

### Study Implications

This study’s findings constitute a valuable novel contribution to knowledge, because they reflect internationally comparable data on parental engagement and parental acceptance/use of technology education, which has previously received limited attention. The pandemic has revealed countless obstacles that parents have been facing daily when seeking to educate their children at home. Regardless of their preparation and skills to support learning, the primary responsibility for enforcing and maintaining young people’s educational engagement lies on the parents.

In sum, this study has provided valuable information regarding the factors that influence how parents accept and use technology: how they are building their IT capacity to support their children at home, their parenting practices assisted by technology, their new partnerships to respond to their new role and challenges, the opportunities as well as the barriers to engaging when deploying educational technology to support children’s learning. The above findings can inform researchers, practitioners, and policymakers in identifying ways to support parental engagement with children’s learning beyond the provision of devices and access.

### Study Limitations

Whilst this study’s results provide valuable insights into how to enhance parental engagement in children’s learning, some limitations should be noted. The analysis in this paper is an all-countries one, where some variables differ from country to country, thus limiting the generalisability of the results. Moreover, data collection was done via an online survey and social networks, thus only parents with access to the internet could provide answers. Finally, the qualitative data were gathered via open-ended questions within the survey, which meant that follow up questioning was not possible.

## Conclusion

This is the first study to report the relationship between technology acceptance and use and parental engagement with children’s learning, by incorporating objective measurement among 4,600 parents from 19 countries during the national lockdowns due to the COVID-19 pandemic. The results have shown that parents are generally struggling with complex educational technology, which (Rogers, 1983, 2003) can act as an obstacle to their engagement with their children’s learning effectively. However, regardless of the difficulties they might encounter while dealing with technology, many manage to engage in the use of technology to support such learning. Further empirical research is needed to examine parental engagement and the educational technology landscape at the country level, including in-depth qualitative research that looks at both schools’ and parents’ perspectives.

Beyond providing devices and access, it is necessary to support families in dealing with educational technology. As this research has shown, perceived complexity in educational technology stops many parents from accepting it, but when they do so, they are able to contribute to their children’s learning. In sum, it is essential that opportunities are provided to parents that help them overcome technological barriers to their engagement in their children’s learning, thereby lessening the achievement gap that has been widening for many during the current pandemic.

## Data Availability Statement

The datasets presented in this study can be found in online repositories. The names of the repository/repositories and accession number(s) can be found below: Journal data in Brief https://doi.org/10.1016/j.dib.2021.106813; Mendeley: https://data.mendeley.com/datasets/kvvdgvs8zs/2.

## Ethics Statement

The studies involving human participants were reviewed and approved by The University of Bath provided ethical approval EIRA1–5408. The patients/participants provided their written informed consent to participate in this study.

## Author Contributions

EMO-S contributed to the conception and design of the study, and wrote the different versions of the manuscript. NE performed the statistical analysis and wrote the quantitative portion of the methods section of the manuscript. AS-H contributed as a supervisor of the study, also revised, read, and approved the draft and the submitted version of the manuscript. All authors contributed to the article and approved the submitted version.

## Conflict of Interest

The authors declare that the research was conducted in the absence of any commercial or financial relationships that could be construed as a potential conflict of interest.

## Publisher’s Note

All claims expressed in this article are solely those of the authors and do not necessarily represent those of their affiliated organizations, or those of the publisher, the editors and the reviewers. Any product that may be evaluated in this article, or claim that may be made by its manufacturer, is not guaranteed or endorsed by the publisher.
